# Physicochemical Properties and Fatty Acid Profiling of Texturized Pea Protein Patties Partially Replaced with Chia Seed Powder During Refrigerated Storage

**DOI:** 10.3390/foods15020270

**Published:** 2026-01-12

**Authors:** Kartik Sharma, Aminee Saree, Ramida Jeenplangchat, Haymar Theinzan, Samart Sai-Ut, Passakorn Kingwascharapong, Supatra Karnjanapratum, Saroat Rawdkuen

**Affiliations:** 1 Unit of Innovative Food Packaging and Biomaterials, School of Agro-Industry, Mae Fah Luang University, Chiang Rai 57100, Thailand; kartik.coa@gmail.com; 2 Innovative Food Science and Technology Program, School of Agro-Industry, Mae Fah Luang University, Chiang Rai 57100, Thailand; 3 Department of Food Science, Faculty of Science, Burapha University, Chonburi 20131, Thailand; samarts@go.buu.ac.th; 4 Department of Fishery Products, Faculty of Fisheries, Kasetsart University, Bangkok 10900, Thailand; passakorn.ki@ku.th; 5 Faculty of Agro-Industry, Chiang Mai University, Chiang Mai 50200, Thailand; supatra.ka@cmu.ac.th

**Keywords:** amino acid, chia seed, fatty acid profile, plant-based patty, textured pea protein

## Abstract

The increasing demand for sustainable, nutrient-dense plant-based foods has intensified interest in functional ingredients that enhance nutritional quality. This study developed plant-based patties by partially replacing texturized pea protein with chia seed powder (CSP; *Salvia hispanica* L.) and evaluated their quality during 20 days of refrigerated storage (4 °C) under nitrogen-flushed packaging. Six formulations (F1–F6) containing 0–25% CSP were evaluated for physicochemical properties, lipid oxidation, and nutritional composition. Based on an optimal balance of texture, cooking yield, antioxidant capacity, and nutritional enhancement, the formulation containing 20% CSP was selected for further analyses. Proximate analysis revealed significant increases in protein (18–21%), fat (9–12%), and ash (2–3%) contents, accompanied by a slight reduction in moisture. All formulations maintained a stable pH throughout storage. Lipid oxidation increased gradually from 0.10–0.17 to 0.89–1.10 mg MDA/kg over 20 days but remained within acceptable limits. Fatty acid profiling indicated enhanced polyunsaturated fatty acids, particularly omega-3 and omega-6. Amino acid analysis showed elevated levels of key amino acids, including glutamic acid, aspartic acid, arginine, leucine, and lysine. Overall, patties containing 20% CSP exhibited improved nutritional quality and satisfactory oxidative stability, highlighting CSP as a promising functional ingredient for plant-based meat alternatives.

## 1. Introduction

The demand for health-oriented food products is increasing simultaneously with the increase in population. Nowadays, consumers are more inclined to consume snack products such as biscuits, cookies, and other ready-to-cook or ready-to-eat meat-like products, such as patties, rather than complete meals [1]. These foods possess insufficient protein and lack balanced nutrition [2]. In developing nations, the standard diet is predominantly cereal-based and often lacks sufficient high-quality protein, contributing to the prevalence of protein-energy malnutrition (PEM) [3].

In response to these nutritional challenges, food researchers and industries are increasingly focusing on protein enrichment strategies and the development of alternative protein-based foods that can deliver balanced nutrition while maintaining consumer acceptability [4]. Plant-based meat analogs have emerged as promising solutions to address both nutritional inadequacy and sustainability concerns, particularly in regions facing protein scarcity. Recent studies have highlighted that plant-based patties can significantly contribute to dietary protein intake when appropriately formulated with functional and protein-rich ingredients [5].

Proteins, being the building blocks, play a major role in the growth of the body and in repairing tissues [6]. Growing consumer awareness towards environmental sustainability, ethical concerns, and other health-related issues associated with animal-based foods has directed their interest towards plant-based meat products. These products are designed to mimic nutritional, functional and sensorial properties of meat, with the sole use of plant-derived ingredients. Pea, wheat and soy proteins are the primary plant proteins that are employed in the development of high moisture meat analogs [7]. Although soy and wheat remain widely used and offer notable processing advantages, there has been a growing shift in the meat alternative market toward the use of clean-label ingredients. 

This shift toward clean-label formulations is driven by consumer demand for minimally processed foods with recognizable ingredients and reduced allergenicity. Recent reports indicate that clean-label positioning plays a critical role in consumer acceptance of plant-based meat alternatives, influencing both purchasing decisions and perceived product healthfulness [8]. Consequently, the replacement or supplementation of conventional binders and additives with natural ingredients has become an important research focus.

Pea proteins, being both ‘non-GMO’ and low allergen crops, offer unique labelling properties. Furthermore, to enhance their value in the food industry, these proteins are usually texturized to mimic the fibrous structure and whole-muscle appearance of real meat [9]. However, the sole use of textured pea proteins (TPP) in formulations may exhibit several limitations, such as poor binding ability, limited oxidative stability and reduced juiciness over traditional meat products. To address this limitation, various hydrocolloids, dietary fibers, and natural functional ingredients can be utilized to enhance texture, water-holding capacity (WHC), and product stability during storage.

Several recent studies have demonstrated that the incorporation of natural fibers and plant-derived functional ingredients can significantly improve the technological performance of plant-based patties by enhancing moisture retention, texture integrity, and oxidative stability during storage [10]. These ingredients also contribute to improved nutritional quality by increasing dietary fiber content and antioxidant potential, which are often limited in conventional meat analog formulations.

Chia seeds (*Salvia hispanica* L.) have gained considerable interest among researchers as a multifunctional food ingredient due to their rich composition of dietary fibers (up to 35%), proteins (25%), oil (33%), omega-3 fatty acids and phenolic compounds [11]. Dietary fibers possess a neutral flavor and enhance water retention, thereby reducing cooking loss. These soluble fibers are used to modify texture and regulate water migration in meat or meat-mimic products [12]. Chia seed powder (CSP) obtained upon grinding possesses better emulsifying and binding ability and can be used as a thickener or stabilizer in the food industry [12]. In addition, it enhances moisture retention and oxidative resistance in food systems [1]. These characteristics employ the use of CSP in various food formulations, including bread, pound cakes and many more [1]. Despite these advantages, there is a limited study on the incorporation of such ingredients in ready-to-eat products like patties.

Therefore, the present study aimed to develop plant-based burger patties using textured pea protein and different concentrations of chia seed powder and to investigate their impact on physicochemical properties, texture, oxidative stability, antioxidant activity, and nutritional quality during refrigerated storage.

## 2. Materials and Methods

### 2.1. Ingredients and Chemicals

Textured pea protein (TPP) was sourced from Leplants, Bangkok, Thailand. Pea protein isolate (PPI) was purchased from Matell, Bangkok, Thailand and Chia seed powder (CSP) was procured from Healthy Choice Asia Co., Ltd., Bangkok, Thailand. Sodium chloride (NaCl), black pepper (BP), onion powder (OP), garlic powder (GP) and beetroot powder were purchased from local suppliers in Prung Thip, Samut Sakhon, Thailand.

All chemicals used were of analytical grade. Chloroform and ethanol were obtained from RCI Labscan, Bangkok, Thailand. 2-Thiobarbituric acid and cumene hydroperoxide were purchased from Fluka Co. (Buchs, St. Gallen, Switzerland). Sodium hydroxide and methanol were procured from Merck (Darmstadt, Germany). The rest of the chemicals were acquired from Sigma Aldrich, Inc. (St. Louis, MO, USA).

### 2.2. Preparation of Textured Pea Protein and Chia Seed Powder

Textured pea protein (TPP) was hydrated in water at 1:4 (*w*/*v*) for 15 min, followed by the addition of CSP, pea protein isolate (PPI), methyl cellulose (MC) and seasoning as shown in Table 1. The mixtures were mixed thoroughly using a blender at 12,000 rpm for 1 min. A total of six formulations were prepared by incorporating CSP at different concentrations (10%, 15%, 20%, and 25%) through partial replacement of TPP. The formulation without CSP and MC served as a negative control (F1), while the formulation without CSP served as a positive control (F2). The prepared mixtures were shaped into patties (113 g each) using a circular mold (8.5 cm diameter, 2 cm thickness).

The patties were then cooked in an air fryer at 180 °C for 5 min on each side to ensure proper cooking. The samples had a thickness of 1.23–1.40 cm, a diameter of 8–8.3 cm, and a weight of 90–111 g (Table 2). The samples were packed in polyethylene (PE)-laminated bags under modified atmosphere packaging (MAP) with 100% nitrogen flushing and sealed. The samples were stored at 4 °C and analyzed on Day 0 and subsequently at 4-day intervals for up to 20 days of storage.

### 2.3. Analyses of Plant-Based Patties

#### 2.3.1. Proximate Composition and Chemical Properties

Proximate composition of plant-based patties was analyzed following the protocol of the AOAC method using analytical numbers 928.08, 960.39, 920.152, and 950.46 for moisture, ash, fat and protein contents, respectively [13]. Moisture content was determined by drying the sample in a hot air oven at 105 °C until constant weight was achieved. Ash content was measured by incinerating the samples at 550 °C for 8 h in a muffle furnace. Fat content was analyzed by heating the dried samples and subsequently extracting lipids with petroleum ether in a Soxhlet apparatus. Protein content was quantified by determining total nitrogen through the Kjeldahl method, applying a conversion factor of 6.25.

Total fiber content was measured using the enzymatic–gravimetric method outlined in AOAC Official Method 985.29 [14]. Briefly, 1 g of defatted and dried sample was subjected to enzymatic digestion using α-amylase (95–100 °C) to hydrolyze starch. This was followed by digestion with protease (pH 7.5, 60 °C) and amyloglucosidase (pH 4.5, 60 °C) to eliminate proteins and residual starch. After enzymatic hydrolysis, 95% ethanol was added to precipitate the soluble fiber fraction. The resulting residue was then separated by filtration or centrifugation, washed with ethanol and acetone, dried at 105 °C, and weighed. The final fiber content was calculated by subtracting ash and residual protein.

Iron content was determined as per the method of [14] using atomic absorption spectrophotometry (AAS). Briefly, 5 g of the sample was dried in a hot air oven at 105 °C and ashed in a muffle furnace until a light grey ash was obtained. Thereafter, the ash was dissolved in concentrated HCl, and the volume was made to 50 mL using deionized water. Iron concentration was measured using AAS equipped with an air-acetylene flame, operating at a wavelength of 248.3 nm. Quantification was performed using external calibration with standard iron solutions prepared from certified stock solutions. Each sample was analyzed in triplicate, and the results were expressed as mg Fe per 100 g sample.

DPPH radical scavenging activity was determined as per the protocol outlined by [15,16]. Briefly, 4 g of the sample was mixed with 20 mL of methanol. The mixture was mixed vigorously and allowed to stand at room temperature in the dark for 30 min. The absorbance of the resulting solution was measured at 517 nm using a spectrophotometer (Thermo Scientific, Waltham, MA, USA, GENESYS 30). The blank was prepared in the same manner, except that distilled water was used instead of the sample. A standard curve was prepared using Trolox, and the activity was expressed as μmol Trolox equivalents (TE)/g.

Protein profiles of six different formulations of patty samples were determined by using SDS PAGE by mixing with buffer solution at a 1:1 ratio. The buffer solution was prepared by mixing 0.5 M Tris-HCL (pH 6.8) with 4% SDS, 20% glycerol and 0.03% Bromophenol Blue with/ without 10% DTT. The mixer and buffer solution were boiled for 3 min. Protein-containing samples were loaded into 4–20% Roti-PAGE Gradient precast gels, followed by running at 60 mA constant current using a Biostep^®^ GmbH power supply (Jahnsdorf, UK). The electrophoresis buffer tank was filled with a buffer solution. The gels were stained overnight in a staining solution (Coomassie Blue R-250-methanol-acetic acid) and shaken gently at 50 rpm. De-staining solution I and II (methanol–acetic acid and water) were used to de-stain gels until the background was clean. Finally, the gels were dried.

#### 2.3.2. Physicochemical and Cooking Characteristics

Water activity (a_w_) of patties was measured using a water activity meter (AquaLab CX-3TE, Decagone Devices, Inc., Pullman, WA, USA).

The color of patties was determined using a LabScan XE colorimeter (HunterLab, Reston, VA, USA). The instrument was calibrated before each set of measurements using standard white, black, and green reference tiles. All the measurements were taken in triplicate, and the values were expressed as L* (lightness), a* (greenness-redness), and b* (blueness-yellowness) [17].

The hardness (kg), chewiness (g × mm), springiness, and cohesiveness of plant-based patties were measured using a texture analyzer (TA. XT plus, Stable Micro Systems, Godalming, UK) equipped with a cylindrical probe (35 mm diameter). A double compression test, simulating chewing, was performed to measure the hardness, chewiness, and springiness of the plant-based patties. The test speed was set at 1 mm/s, and the samples were compressed to 50% of their original height. All measurements were taken in triplicate at room temperature to ensure consistency, and the average values were reported.

The water holding capacity (WHC) of the plant-based patty was measured using the method described by Sheng [18] with slight modifications. Briefly, a 2% (*w*/*v*) dispersion was prepared in distilled water and stirred overnight. The mixture was then centrifuged at 20,000× *g* for 30 min to separate the supernatant and pellet. The wet pellet was collected, weighed and subsequently oven-dried at 105 °C overnight. After drying, the pellet was weighed again. The WHC was calculated using the following equation,

$$WHC=\frac{\left(M1\right)-(M2)}{\left(M2\right)}$$

where *M*1 = Mass of wet pallet; *M*2 = Mass of dry pallet.

Cooking loss was evaluated as per the protocol outlined by [19]. Briefly, the weight difference between the raw and cooked chicken patties was recorded, and cooking loss was calculated and expressed as grams per 100 g of sample.

#### 2.3.3. Storage Stability of Plant-Based Patties During Refrigeration

The protocol outlined by Sharma et. al. [19] was used to measure the pH of the patties with slight modifications. Briefly, 10 g of patty was mixed in 50 mL of distilled water. The mixture was homogenized at 20,000 rpm for 1 min. The pH of the homogenate was recorded using a pH meter (Orion Star A211, Thermo Scientific, Waltham, MA, USA).

Lipid oxidation was monitored in plant-based patties using thiobarbituric acid reactive substances (TBARS) assay as detailed by Jeong et al. [20]. Briefly, 10 g of the sample was homogenized in 7.5% trichloroacetic acid (50 mL), followed by filtration using filter paper. 5 mL of this filtrate was transferred to tubes containing 5 mL of 0.22 M of TBARS. The mixture was allowed to heat in a water bath at 100 °C for 15 min. The mixture was then cooled using an ice bath. An absorbance at 532 nm was measured using a spectrophotometer. A standard curve of malonaldehyde was prepared as stated above, and the value was expressed as milligrams of MDA equivalents per kg of samples.

Based on a comparative evaluation of physicochemical composition, cooking loss, texture parameters, antioxidant activity, and nutritional enhancement, the best formulation will be selected for detailed storage stability, amino acid, and fatty acid analyses, along with a positive control (F2).

Microbiological analysis was conducted as per the protocol outlined by Sharma et al. [19]. The patties incorporated without and with CSP were analyzed for microbiological quality. A total of 25 g of each sample was aseptically transferred to a Stomacher bag with 225 mL of 0.1% sterile peptone water and was homogenized using a Stomacher for 2 min. Ten-fold serial dilutions were prepared. Each sample with appropriate dilution (1 mL) was spread on plate count agar (PCA) for the enumeration of total viable count (TVC), psychrophilic bacteria counts (PBCs), followed by incubation at 37 °C for 24 h and 4 °C for 7 days, respectively. For the determination of *Enterobacteriaceae*, 100 μL of a suitable dilution from each sample was spread on eosin methylene blue agar plates (HiMedia Laboratories LLC, Mumbai, India) and incubated at 37 °C for 24 h. Lactic acid bacteria were detected by spreading the sample with selected dilution on deMan-Rogosa Sharpe agar (MRS) (Oxoid, Thermo Fischer Scientific, Waltham, MA, USA), and the plates were incubated at 37 °C for 48 h. Moreover, *Pseudomonas* spp. was determined using Pseudomonas isolation agar (Oxoid, Thermo Fischer Scientific, Waltham, MA, USA) supplemented with glycerol and incubated at 37 °C for 48 h. After incubation, colonies from each plate were counted, and microbial loads were expressed as log CFU/g.

#### 2.3.4. Nutritional Profiling of Selected Plant-Based Patties

Amino acid composition of the selected patties was analyzed by Central Laboratory Co., Ltd. (Chiang Mai, Thailand) following the in-house TE-CH-372 analytical protocol based on the Official Journal of the European Communities, L257/16. The results were expressed as mg per 100 g of the sample.

Gas chromatography equipped with a flame ionization detector (GC-FID) was used to measure the fatty acid profile of the selected patties by Central Laboratory Co., Ltd. (Chiang Mai, Thailand) following the in-house TE-CH- 372 analytical protocol based on [13], Method 996.01. Briefly, lipid extracts from patties were converted to fatty acid methyl esters (FAMEs) and subsequently injected into a gas chromatograph (Agilent Model 6890N, G1530N, Serial No. US10406046, Santa Clara, CA, USA) equipped with a Supelco SP-2560 capillary column. The injector and detector temperatures were set at 250 °C and held for 17 min, with a total run time of 55 min. FAMEs were identified against authentic fatty acid standards and quantified using internal standard calibration. Results were expressed as a percentage of total identified fatty acids in g/100 g of sample.

### 2.4. Statistical Analysis

All experiments were conducted in triplicate (*n* = 3), and results are expressed as mean ± standard deviation. Data were analyzed using one-way analysis of variance (ANOVA). When significant differences were detected, Duncan’s multiple range test was applied for post hoc mean comparison. Student’s *t*-test was used only for pairwise comparisons between two selected samples where applicable. Differences were considered statistically significant at *p* < 0.05. Statistical analyses were performed using SPSS software (version 11.0, SPSS Inc., Chicago, IL, USA).

## 3. Results and Discussion

### 3.1. Chemical Composition and Chemical Properties of Plant-Based Patties

#### Proximate Analysis

The proximate composition of plant-based patties fortified without and with CSP is shown in Table 2. Moisture content in plant-based patties ranged from 53% (F6) to 75% (F1). A significant reduction in the moisture content was observed with progressive replacement of TPP by CSP (*p* < 0.05). The negative control (F1) showed the highest moisture content (*p* < 0.05). This reflects the greater proportion of hydrated protein and lower total solids. In contrast, CSP-containing patties exhibited lower moisture contents, with the least moisture content in the patty having the highest CSP levels (*p* < 0.05). This significant decrease might be attributed to compositional and functional effects of CSP over TPP. Replacement of hydrated TPP by dried CSP results in increased solid content and reduces the amount of added water. In addition, the mucilage fractions and soluble fibers of CSP bind water within their dense matrix, thereby limiting the proportion of free, extractable moisture after cooking. Senna et al. [1] and Mishra et al. [21] also observed the reduced moisture content in various plant-based and fiber-enriched burgers. The addition of whole chia flour alters the protein matrix, making it more compact and enhancing its capacity to retain water molecules. The presence of fiber components also contributes to increased total solids and reduced free water availability, plausibly due to entrapment of water within the structural matrix. Furthermore, chia-derived emulsions or chia flour have been documented to minimize water loss and alter moisture distribution in both meat and plant-based analogs [22].

The protein content in patties ranged from 18% to 24%, with the highest protein content in patties with the highest concentration of CSP (*p* < 0.05) (Table 2). A significant increase in protein content was observed with the progressive replacement of TPP by CSP (*p* < 0.05). The lowest protein content observed in the positive control (F2) (*p* < 0.05) was associated with the higher moisture and dilution effect of hydrated TPP. In contrast, the higher protein content in CSP-containing patties was attributed to the nutritional composition of CSP. Generally, chia seeds contain up to 25% of high-quality protein with a balanced amino acid profile [1,23]. Therefore, increased CSP inclusion resulted in an augmented level of solid mass in the mixture, leading to an apparent elevation in protein concentration after cooking due to reduced water retention. Chia seed protein provides textural firmness owing to its high content of glutamic acid and aspartic acid, which interact with polysaccharides and water molecules to stabilize the matrix.

The ash content in formulated plant-based patties ranged from 1.69% to 2.48%. A significant increase in ash content was observed with increased concentration of CSP in patties (*p* < 0.05). Chia seeds, being rich in mineral composition, particularly calcium (in abundance, 460–671 mg/100 g), magnesium (250–322 mg/100 g), and phosphorus (393–661 mg/100 g), including other trace elements like iron and zinc, was plausibly responsible for increased ash content in the formulations containing CSP (F3–F6) [24]. Ashura et al. [23] also reported the higher ash values in chia seeds ranging between 4–6%. Therefore, the substitution of TPP by CSP not only elevated the total solid content but also introduced these intrinsic minerals into the patty matrix, leading to higher residual ash following moisture removal.

The fat content ranged from 1.4% to 8.4% (Table 2). A significant increase in fat content was observed with the progressive replacement of TPP by CSP (*p* < 0.05). The control samples (F1 and F2) exhibited the lowest fat values due to the absence of lipid-rich ingredients, whereas patties containing CSP showed a consistent rise in fat content, reaching the maximum value in F6, which incorporated the highest level of CSP (*p* < 0.05). The augmented levels of fat content can be directly attributed to the intrinsic lipid composition of chia seeds. Chia seeds usually contain 15–35% fat content. It is a rich source of polyunsaturated fatty acids (PUFA), primarily consisting of α-linolenic acid or omega-3 (>60%) and linoleic acid or omega-6 (>20%) [23]. Chia seed oil serves as a rich, plant-derived source of omega-3 fatty acids and is frequently marketed as a vegan substitute for fish oil, providing benefits related to cardiovascular health and inflammation management [25].

The fiber content of the formulated patties also increased significantly with the addition of CSP, ranging from 1.3–16% (*p* < 0.05). The control samples (F1 and F2) exhibited the lowest fiber contents. This was due to the absence of an additional fiber source present in the control formulations except for TPP. In contrast, the CSP-fortified formulations (F3–F6) demonstrated a consistent and marked rise in total fiber. This was directly related to the augmented concentration of CSP from F3 to F6. Chia seeds contain approximately 34–36% total dietary fiber, composed mainly of insoluble fibers (cellulose, hemicellulose) and soluble mucilage polysaccharides, both of which substantially elevate the fiber content of composite products [26].

Overall, the substitution of TPP by CSP significantly altered the nutritional and functional balance of the plant-based patties, producing formulations with lower moisture and higher protein, fat, fiber, and ash contents.

For the iron content, the control samples (F1 and F2) showed comparable values, indicating the negligible impact of the addition of methyl cellulose on mineral content. However, inclusion of CSP at 10–20% (F3–F5) resulted in lower detectable iron levels, likely due to binding of iron by dietary fibers and other phytochemicals present in chia. At further inclusion of CSP (25% in F6), the intrinsic iron contribution of CSP appeared to outweigh these interactions, leading to the highest measured iron content among all formulations (*p* < 0.05). Therefore, the moderate inclusion of CSP reduced the extractable iron content due to metal-binding phytochemicals, whereas higher incorporation (25%) contributed enough intrinsic minerals to significantly increase total iron levels in the patties.

### 3.2. Physicochemical and Cooking Characteristics

#### 3.2.1. Water Activity

The water activity (a_w_) of the plant-based patties ranged from 0.95 (F1) to 0.91 (F6), showing a significant reduction with increasing inclusion of CSP (*p* < 0.05) (Table 2). The highest a_w_ was observed in the samples (F1 and F2), reflecting a greater proportion of unbound moisture associated with hydrated TPP. In contrast, CSP-fortified patties showed lower a_w_ values, indicating the immobilization of water molecules within the matrix. This might be due to the high-water binding capacity of chia mucilage and soluble fibers that bound the available free water, resulting in reduced a_w_. Senna et al. [1] also documented the strong water-binding and gel-forming capacity of chia components. It was reported that mucilage and soluble fibers in CSP exhibit high water absorption and form 3-D networks that immobilize the water. This phenomenon decreases the fraction of water available for microbial growth and deteriorative reactions [1]. Various other studies on burger or similar products also documented that the use of fibers and hydrocolloids promotes water stabilization, resulting in lower a_w_ and improved storage stability [18,27,28]. Overall, the reduction in a_w_ in CSP-formulated patties (F3–F6) in comparison to control (F1 and F2) aligns with their lower moisture levels and increased fiber and solid content. Therefore, CSP influences the overall composition by immobilizing water within the matrix and reducing the amount of free, available water, which may contribute to the extended shelf life of plant-based patties.

#### 3.2.2. Color

**Figure 1:**
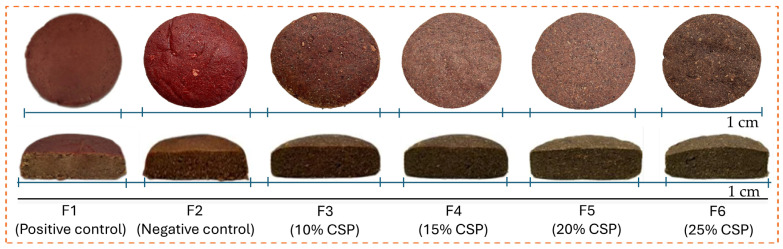
Surface and cross-sectional images of cooked plant-based patties formulated with different concentrations of chia seed powder. Images are representative of patties prepared from three independent batches. Scale bar = 1 cm.

The color attributes (L*, a* and b*) of plant-based patties are presented in Table 3 and visually illustrated in Figure 1. A significant decrease in lightness (L*) and redness (a*)-values was observed upon substitution of TPP by CSP (*p* < 0.05). Notably, control patty (F1) exhibited the highest values of L* and a*-values owing to reddish brown color of the patties, as can be seen in Figure 1. Addition of MC, particularly in positive control (F2), slightly reduced the L*-values in patties while maintaining similar a*-values, resulting in deeper surface tone. As TPP was gradually replaced by CSP, a general darkening effect was observed, particularly up to F5. The L* and a* values decreased significantly (*p* < 0.05) from F1 to F5, indicating a transition from bright reddish-brown (F1–F2) to deeper brown hues (F3–F5). This can be attributed to the darker color of CSP and its high fiber and phenolic content, which absorb and scatter light more strongly than hydrated TPP.

However, the upsurge in L*, a*, and b*-values in F6 suggests surface lightning and pigment oxidation at the highest CSP level. The augmented concentration of CSP in F6 led to reduced surface moisture (as discussed above), which might have resulted in browning and increased light reflectance following air frying. Furthermore, the decrease in a* and b*-values in F3 to F5 was plausibly due to dilution of pigments from beet root and intensified Maillard reactions. On the other hand, the slight upsurge in F6 could be the result of surface carbohydrate caramelization or oxidative changes in polyphenolic constituents. Comparable trends have been documented in chia-substituted chicken sausages, where increasing chia levels (5–15%) significantly decreased L* (62.76 to ~41), lowered b* (16.14 to ~13.53), and increased a* (1.11 to ~3.95), attributed to the dark hue of chia flour and heat-driven pigment–protein interactions (chicken-sausage CSP study) [12]. These outcomes are parallel with the results of our study and support the idea that CSP drives a transition from bright reddish-brown (F1–F2) toward deeper brown tones (F3–F5), with a slight surface-lightening/reflectance shift at the highest inclusion (F6). At higher inclusion levels, CSP contributed to uniform browning and surface reflectance typical of fiber-rich plant analogs.

#### 3.2.3. Textural Properties

The textural characteristics of plant-based patties are given in Table 2. Hardness in patties ranged from 2.28 kg (F1) to 7.95 kg (F5), showing a significant increase (*p* < 0.05) with the incorporation of CSP. The control (F1) exhibited the lowest hardness due to its higher moisture and the softer, hydrated matrix formed by TPP. Incorporation of CSP progressively increased the firmness as the fiber-rich chia matrix absorbed water and compacted the structure. However, a slight reduction in hardness for F6 (5.74 kg) suggests that excessive CSP disrupted network uniformity and reduced structural continuity.

No differences were observed in chewiness between the control and the patties containing CSP (*p* > 0.05). The higher variability in certain texture measurements reflects the intrinsic heterogeneity of fiber-enriched plant-based patties. This states that the CSP–TPP combinations did not markedly alter the energy required to deform and masticate the patties. The higher Springiness values differed significantly only for the negative control (F1) (*p* < 0.05). The control (F1) showed the highest elasticity (0.42), while all other treatments remained statistically insignificant (*p* > 0.05). Cohesiveness also declined moderately from 0.34 (F1) to 0.24–0.30 (F3–F6), reflecting weaker internal bonding as insoluble chia fibers partially replaced the hydrated protein structure. These changes could be attributed to the combined effects of functional ingredients in the formulation. The addition of MC in formulations except F1 acts as a thermo-gelling binder that contributes to matrix integrity during heating. When combined with PPI, MC reinforces gel formation upon heating, increasing firmness. Meanwhile, CSP’s mucilage and insoluble fibers immobilize water and add bulk, further tightening the matrix. However, at higher concentrations, CSP’s dominance reduces protein–protein interactions and elasticity.

Overall, these results confirm that the combined action of CSP, MC, and TPP improved firmness and structural stability, creating a denser, meat-like texture. However, excessive CSP inclusion reduced elasticity and cohesiveness, suggesting that an optimal balance between hydrated protein and fiber-bound solids is essential to maintain desirable textural quality in plant-based patties.

#### 3.2.4. Water Holding Capacity

The water holding capacity (WHC) of the patties decreased significantly with the increase in concentration of CSP (*p* < 0.05) (Table 2). The highest WHC in the control (F1) is attributed to the hydrated TPP, which forms a gel network capable of retaining water under compression. With progressive replacement of TPP by CSP (F3–F6), the WHC declined steadily, reflecting a denser, high-solid matrix with less free water to be retained. Although chia-based ingredients exhibit high intrinsic water-binding capacity, much of this water is tightly bound within mucilage and fiber domains and is not released under centrifugation. As CSP levels increased, a larger fraction of water became immobilized in these structures, which reduced the measurable WHC of the cooked patties. Similar trends have been reported by Ishaq et al. [29] and Zhang et al. [30], who noted that excess fiber in plant-based meat analog systems reduces WHC by limiting water mobility.

#### 3.2.5. Cooking Loss

Cooking loss of formulated patties decreased significantly with increased inclusion of CSP (*p* < 0.05) (Table 2). The control patties (F1) exhibited the highest losses, primarily due to their higher moisture and weaker structural integrity. This might have allowed greater drip and fat loss during air frying. In contrast, the incorporation of CSP and MC markedly reduced cooking loss. MC acts as a thermo-gelling binder that forms a stable gel upon heating, reducing drip loss, while CSP’s mucilage and insoluble fibers enhance moisture and fat entrapment within the protein matrix. Chen and others also reported the reduced cooking losses upon the addition of MC in the burger patty. This reduced cooking loss was attributed to the thermal gelation of MC, which enhanced the binding of moisture [31]. Furthermore, it was also reported that MC in food formulations might form an adhesive layer during heating, which can be used as a barrier to prevent water loss [32]. 

Overall, the reduced cooking loss in CSP-containing patties demonstrates the beneficial role of chia-derived fibers and mucilage in enhancing water and fat retention, resulting in improved yield and textural stability after cooking.

#### 3.2.6. Antioxidant Activity (DPPH-RSA)

The antioxidant activity of plant-based patties varied significantly (*p* < 0.05) among formulations, ranging from 59.55% (F2) to 88.58% (F6) (Table 2). The control samples (F1 and F2) exhibited the lowest radical scavenging activity, reflecting the absence or minimal inclusion of antioxidant-rich ingredients. In contrast, the incorporation of CSP markedly enhanced DPPH-RSA values. This improvement is attributed to the high content of phenolic acids, flavonoids, and α-linolenic acid in chia seeds, which are known to scavenge free radicals and inhibit lipid oxidation. Gebremeskal et al. [33] also reported the high total antioxidant activity of chia seeds, particularly due to the presence of high concentrations of flavonoids. This activity was higher than that present in flax seeds and psyllium husk. CSP contains compounds such as caffeic, rosmarinic, and chlorogenic acids, which contribute to strong antioxidant potential.

Furthermore, the mucilage and protein–polyphenol interactions in chia matrices may enhance the stability and availability of antioxidants during processing. Similar trends were reported by Silva and others, who observed increased antioxidant activity in chia-enriched plant-based meat analog products due to elevated levels of bioactive compounds [27].

Overall, the progressive increase in antioxidant capacity with CSP incorporation demonstrates its functional role not only as a structural and textural enhancer but also as a natural antioxidant component that may improve product stability and nutritional quality.

#### 3.2.7. Protein Patterns

**Figure 2:**
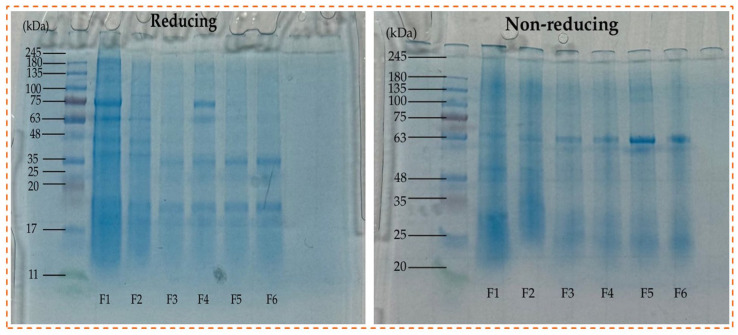
Electrophoresis profile of protein profiles of different burger patties and protein sources. F1—negative control; F2—positive control; F3—10% CSP; F4—15%CSP; F5—20% CSP; F6—25% CSP.

Protein pattern using SDS-PAGE depicts the structural protein content of six patties under reducing (L) and non-reducing (R) conditions, as shown in Figure 2. All formulations exhibited comparable major protein bands, indicating that the primary protein fractions were largely preserved across samples. Prominent bands observed at approximately 20–25 kDa, 35 kDa, and 63–75 kDa correspond to the acidic and basic polypeptides of glycinin (11S globulin) and the α′-, α-, and β-subunits of β-conglycinin (7S globulin), which are characteristic of pea protein.

Under reducing conditions, F1 exhibited the highest band intensities, while CSP-containing formulations (F3–F6) exhibited comparatively weaker bands, suggesting reduced extractable soluble protein or increased aggregation during processing. The attenuation of high-molecular-weight bands upon reduction indicates the dissociation of disulfide-linked protein complexes. In contrast, non-reducing gels revealed the persistence of higher-molecular-weight bands (≥75 kDa), particularly in CSP-enriched samples, indicating the presence of intact protein aggregates stabilized by disulfide and non-covalent interactions.

The enhanced aggregation observed in CSP-containing patties may be attributed to interactions between pea proteins and chia components, including chia proteins, mucilage, and fiber, which can promote protein crosslinking and limit electrophoretic mobility. Overall, the electrophoretic patterns demonstrate that formulation and processing influence protein aggregation behavior, with disulfide bonding playing a key role in structuring the protein network within plant-based patties.

### 3.3. Storage Stability of Plant-Based Patties During Refrigeration

#### 3.3.1. pH Changes

**Figure 3:**
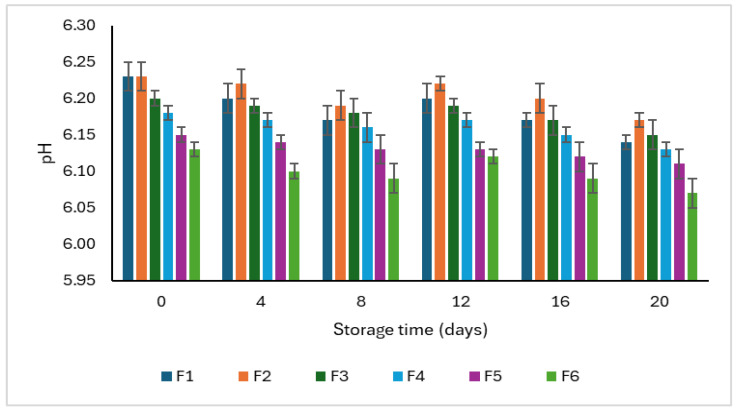
Changes in pH of plant-based patties formulated with varying levels of chia seed powder during 20 days of refrigerated storage (4 °C).

The variation in pH of plant-based patties formulated with different concentrations of CSP during 20 days of refrigerated storage is given in Figure 3. Significant differences were observed among the formulations on each day (*p* < 0.05); however, the pH values exhibited only minor numerical reductions (≤0.1 units) with very small standard deviations (≤0.02), suggesting that these changes were not statistically significant over time.

At day 0, pH values ranged from 6.13 (F3) to 6.23 (F1 and F2). The value gradually declined to 6.07 (F6) and 6.14 (F1) on the 20th day of storage. This slight decrease in pH reflects limited acid formation during cold storage, likely due to minimal microbial metabolism and the gradual production of weak organic acids such as lactic and acetic acids [19]. Notably, all formulations maintained pH values within the stable range (6.0–6.3) commonly reported for legume-protein-based or hydrocolloid-stabilized meat analogs, indicating no spoilage or undesirable fermentation occurred. Zhang and his researchers also documented the stable pH range for legumes, which lies near neutral pH [34]. The stability of plant-based patties could be attributed to the controlled storage conditions and modified atmospheric packaging used in this study. The packaging using nitrogen flushing and storage at 4 °C effectively minimized oxygen availability and microbial proliferation. Such anaerobic, low-temperature environments restrict the growth of spoilage and acid-producing bacteria, thereby limiting the formation of organic acids responsible for pH decline [19].

The formulations with higher CSP concentrations, particularly F5 and F6, exhibited slightly lower pH values than the controls (F1–F2), plausibly due to the mildly acidic nature of chia seed constituents, comprising phenolic acids and soluble polysaccharides, which can impart weak acidity. Additionally, the presence of high fiber and mucilage in chia seeds not only contributes to water binding but also reduces the availability of free water for microbial growth. This also supports pH stability upon storage.

In a nutshell, the pH of all patties remained stable throughout 20 days of refrigerated storage, with minor formulation-dependent differences and no statistically significant effect of time.

Color parameters and visual appearance were evaluated at Day 0 only, as the patties were stored under nitrogen-flushed packaging at 4 °C, conditions known to minimize pigment oxidation and color degradation; therefore, no pronounced visual changes were expected during refrigerated storage.

#### 3.3.2. Lipid Oxidation

**Figure 4:**
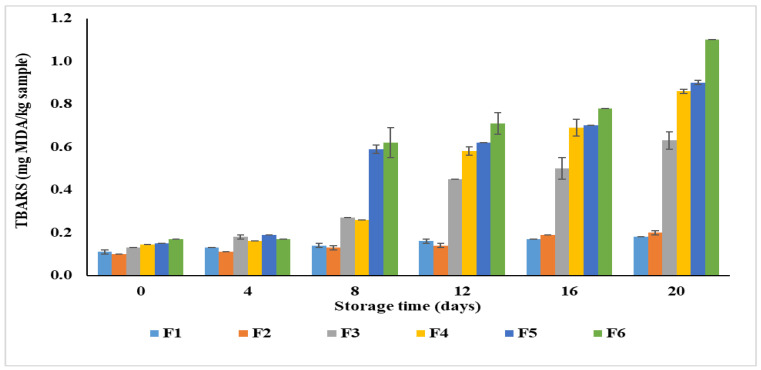
Changes in lipid oxidation (TBARS values) of plant-based patties formulated with varying levels of chia seed powder during 20 days of refrigerated storage (4 °C).

The extent of lipid oxidation in plant-based patties formulated with varying levels of CSP during 20 days of refrigerated storage (4 °C) was assessed using TBARS, as shown in Figure 4. The initial TBARS values ranged from 0.10 mg MDA/kg (F2) to 0.17 mg MDA/kg (F6), indicating minimal lipid oxidation during the onset of storage. As storage progressed, a gradual increase in TBARS values was observed in all formulations (*p* < 0.05). This indicates the progressive formation of secondary lipid oxidation products such as malondialdehyde (MDA). The TBARS value reached 0.18 (F1) and 1.11 mg/kg (F6) after 20 days of storage. Notably, the formulations enriched with CSP exhibited higher TBARS values throughout storage. This might be attributed to the presence of elevated levels of PUFA in CSP-formulated patties, which are more prone to oxidative degradation. The increase was most pronounced in F6 formulation, which had the highest concentrations of CSP and consequently showed the greatest extent of lipid oxidation.

The gradual increase in TBARS values reflects the formation of MDA and other secondary products derived from lipid peroxidation during storage. However, all formulations remained within the acceptable oxidative stability limit (<2 mg MDA/kg) commonly reported for refrigerated meat and meat-analog products. However, there is no legislative TBARS limit for patties. The literature commonly uses ~0.5 mg MDA/kg to indicate the onset of oxidation, ~1 mg MDA/kg as a pragmatic acceptability threshold and ~2.0–2.5 mg MDA/kg as the range at which rancid off-flavors become perceptible to consumers [35,36,37].

In the present study, the combination of nitrogen-flushed packaging and cold storage at 4 °C effectively minimized oxygen exposure and suppressed oxidative reactions. This results in a relatively low oxidation rate in the patties upon storage. Furthermore, CSP’s inherent characteristics, such as phenolic acids, flavonoids, and α-linoleic acid, likely contributed to the inhibition of free radical propagation and partially compensated for the oxidation potential of PUFAs. These results are consistent with the previous studies where chia seed or other plant-based ingredients reduce lipid oxidation in meat or meat analog products. Karpińska et al. [38] also documented the use of chia seeds for reducing the oxidative changes in the pork patties during storage. The lower TBARS values (0.26–0.45 mg MDA/kg product) observed in this study were a result of chia seed’s bioactive compounds content, such as tocopherols, polyphenols and carotenoids, which may be responsible for inhibition of lipid oxidation.

The observed oxidative stability of CSP-fortified patties therefore highlights chia’s dual functionality as both a nutritional enhancer and a natural antioxidant, effectively maintaining product quality during refrigerated storage.

Based on a comparative evaluation of physicochemical composition, cooking loss, texture parameters, antioxidant activity, and nutritional enhancement, formulation F5 (20% CSP) was identified as the most balanced formulation. This formulation exhibited improved firmness without excessive hardness, reduced cooking loss, high antioxidant capacity, and superior nutritional attributes compared to other treatments. Therefore, F5, along with the positive control (F2), was selected for detailed storage stability, amino acid, and fatty acid analyses.

### 3.4. Effect of Storage on Selected Plant-Based Patties

#### Microbiological Properties

Total plate count and total yeast and mold count of plant-based patties incorporated with CSP at varying concentrations during 20 days of refrigerated storage were not detected in any of the patties. This could be attributed to the high temperature (180 °C) used for air frying the patties. This high temperature was capable of successfully reducing the microbial load on the outer and inner surfaces of the patties. Additionally, storage at low temperature (4 °C) simultaneously slowed down the growth of any remaining microorganisms from heating. Generally, most bacteria and molds prefer warmer temperatures for growth, which may result in slow growth and almost no reproduction of the microbes. Therefore, a very low microbial count (below the limit of quantification, i.e., 1 × 10^6^ CFU/g) was detected when patties were cooked in air frying at 180 °C and stored at 4 °C for up to 20 days. Similar results were documented by [19,39], where low bacterial count was observed in chicken wings and chicken patties when cooked in an air fryer for 10 min. Thus, air frying was another potential method to inactivate microorganisms in patties.

### 3.5. Nutritional Profiling of Selected Plant-Based Patties

#### 3.5.1. Amino Acid Profiles

The incorporation of 20% CSP (F5) resulted in a more favorable amino acid profile in comparison to the control sample (F2) formulated solely with hydrated TPP. Since TPP was hydrated at a 1:4 ratio prior to incorporation, its contribution to protein density in the positive control was substantially diluted by water. In contrast, substitution with dry CSP increased the overall total solids and protein proportion per 100 g of finished product. This resulted in higher protein content observed in F5 and the correspondingly elevated amino acid concentrations.

Essential amino acids (leucine, lysine, valine, phenylalanine and threonine) were observed to be higher in F5, due to TPP and CSP’s intrinsic amino acid profile. Chia is known to contain appreciable amounts of tryptophan and arginine, as reflected by elevated levels of these amino acids in F5 (Table 4). On the other hand, the positive control (F2) showed comparatively lower amino acid levels, which might be due to substantial moisture content originating from TPP hydration, which lowered the protein density on a wet basis. However, methionine was detected at low levels in the control sample (<200 mg/100 g protein) but was not detected in the F5 formulation, indicating that methionine remains a limiting amino acid despite the improvement in other essential amino acids, which is consistent with the known amino acid profile of legume- and chia-based protein systems.

Overall, the essential amino acid pattern in F5 was nutritionally superior due to higher concentrations relative to the positive control. These results demonstrate that partial replacement with dry chia seed powder can enhance the amino acid composition of plant-based patties through both higher protein density and complementary amino acid contributions.

#### 3.5.2. Fatty Acid Composition

The fatty acid composition of plant-based patties fortified without and with CSP (20%) is listed in Table 5. Notable differences were observed in the fatty acid profile of control and CSP-added patties. Overall, the fatty acid composition improved significantly in F5 compared with the positive control. CSP, being naturally rich in PUFA, especially α-linolenic acid (ALA; C18:3n3), contributes a favorable omega-3 to omega-6 ratio. Accordingly, F5 exhibited elevated levels of omega-3 and omega-6 fatty acids, along with increased total PUFA content, when compared to the control. In contrast, the positive control showed considerably lower PUFA concentration and reduced omega-3 content. This aligns with the dilute lipid concentration of hydrated TPP. Furthermore, the minimal fat content of hydrated TPP also explains the lower PUFA and omega fatty acid levels observed.

A moderate increase in monounsaturated fatty acids (MUFA), particularly oleic acid, was also observed in F5, supporting the contribution of chia’s intrinsic lipid fraction. Although saturated fatty acids such as palmitic and stearic acid were present in both formulations, their presence was proportionally lower in F5 due to the dominance of PUFA-rich chia lipids. This resulted in a healthier fatty acid profile in F5, characterized by higher PUFA and omega-3 content and a more desirable PUFA–SFA ratio. 

## 4. Conclusions

Chia seed powder (CSP) was successfully used as a functional ingredient to enhance the nutritional quality, physicochemical properties, and overall acceptance of plant-based patties formulated with textured pea protein. CSP incorporation increased protein, dietary fiber, fat, and antioxidant activity while reducing moisture content and water activity, resulting in improved cooking yield and structural firmness. Although higher CSP levels promoted lipid oxidation due to increased polyunsaturated fatty acids, oxidative stability remained within acceptable limits during refrigerated storage. Among all formulations, patties containing 20% CSP achieved the most balanced combination of texture, nutritional value, and fatty acid composition based on comprehensive physicochemical and nutritional properties. These findings indicate that optimized CSP inclusion offers a clean-label strategy for developing stable, nutritious, and appealing plant-based meat alternatives.

## Figures and Tables

**Table 1:** Formulation composition of plant-based patties prepared with different levels of chia seed powder (CSP).

Formulation	TPP (Hydrated)	CSP (g)	CSP (%)	MC (g)	PPI (g)	NaCl (g)	BP (g)	OP (g)	GP (g)	BRP (g)
F1 (Negative control)	104	-	0	-	4.00	0.50	0.80	1.00	1.00	1.70
F2 (Positive control)	100	-	0	4.00	4.00	0.50	0.80	1.00	1.00	1.70
F3	88.70	11.30	10	4.00	4.00	0.50	0.80	1.00	1.00	1.70
F4	83.05	16.95	15	4.00	4.00	0.50	0.80	1.00	1.00	1.70
F5	77.40	22.60	20	4.00	4.00	0.50	0.80	1.00	1.00	1.70
F6	71.75	28.25	25	4.00	4.00	0.50	0.80	1.00	1.00	1.70

Note: Values are expressed as grams (g), calculated based on a total formulation weight of 113 g. TPP values represent the hydrated weight after soaking at a 1:4 (*w*/*v*) TPP to water ratio.

**Table 2:** Proximate composition, physicochemical, textural, and antioxidant properties of plant-based patties formulated with different levels of chia seed powder.

	F1	F2	F3	F4	F5	F6
Moisture content (%)	75.5 ± 0.2 ^a^	72.5 ± 0.3 ^b^	64.5 ± 0.2 ^c^	60.6 ± 0.2 ^d^	54.9 ± 0.6 ^e^	53.4 ± 0.0 ^f^
Ash (%)	1.7 ± 0.0 ^e^	1.7 ± 0.0 ^e^	2.1 ± 0.1 ^d^	2.2 ± 0.0 ^c^	2.3 ± 0.0 ^b^	2.5 ± 0.0 ^a^
Protein (%)	19.3 ± 0.6 ^d^	18.2 ± 0.2 ^e^	18.4 ± 0.4 ^e^	20.1 ± 0.4 ^c^	22.3 ± 0.6 ^b^	23.9 ± 0.2 ^a^
Fat (%)	1.5 ± 0.1 ^e^	1.5 ± 0.1 ^e^	3.6 ± 0.3 ^d^	5.4 ± 0.2 ^c^	6.4 ± 0.4 ^b^	8.4 ± 0.2 ^a^
WHC	2.3 ± 0.2 ^a^	1.2 ± 0.1 ^b^	0.8 ± 0.0 ^c^	0.8 ± 0.0 ^d^	0.6 ± 0.1 ^e^	0.5 ± 0.0 ^e^
Iron (mg/100g)	10.7 ± 0.0 ^b^	10.4 ± 0.0 ^b^	9.1 ± 0.1 ^c^	9.9 ± 0.0 ^c^	9.9 ± 0.0 ^c^	11.1 ± 0.0 ^a^
Water activity (a_w_)	0.98 ± 0.0 ^a^	0.98 ± 0.0 ^a^	0.97 ± 0.0 ^a,b^	0.97 ± 0.0 ^b^	0.97 ± 0.0 ^b^	0.97 ± 0.0 ^b^
Fiber (%)	1.3 ± 0.0 ^e^	2.0 ± 0.6 ^e^	6.2 ± 0.3 ^d^	9.3 ± 1.0 ^c^	12.6 ± 0.4 ^b^	15.9 ± 0.6 ^a^
Cooking loss (%)	16.0 ± 0.7 ^a^	14.5 ± 0.9 ^b^	9.9 ± 0.7 ^c^	9.1 ± 0.4 ^c^	8.9 ± 0.1 ^c^	7.9 ± 0.3 ^d^
Thickness	1.2 ± 0.1 ^b^	1.4 ± 0.1 ^a,b^	1.4 ± 0.1 ^a^	1.4 ± 0.1 ^a,b^	1.3 ± 0.1 ^a,b^	1.40 ± 0.1 ^a^
Diameter (mm)	8.3 ± 0.2 ^a^	8.1 ± 0.1 ^a,b^	8.0 ± 0.0 ^b^	8.2 ± 0.2 ^a,b^	8.1 ± 0.1 ^a,b^	8.1 ± 0.1 ^a,b^
Weight (g)	100.7 ± 0.5 ^c^	89 ± 1.0 ^d^	106 ± 1.0 ^b^	105.3 ± 0.6 ^b^	109.7 ± 0.6 ^a^	110.3 ± 0.6 ^a^
Textural properties						
Hardness (kg)	2.3 ± 0.4 ^d^	7.3 ± 0.3 ^a,b^	6.2 ± 0.6 ^c^	6.5 ± 0.5 ^b,c^	7.9 ± 0.6 ^a^	5.7 ± 0.7 ^c^
Chewiness	325.0 ± 126.5 ^a^	552.5 ± 196.7 ^a^	431.2 ± 155.9 ^a^	478.6 ± 31.2 ^a^	487.3 ± 28.2 ^a^	529.3 ± 114.8 ^a^
Springiness	0.4 ± 0.1 ^a^	0.3 ± 0.0 ^b^	0.3 ± 0.0 ^b^	0.3 ± 0.0 ^b^	0.2 ± 0.0 ^b^	0.3 ± 0.1 ^b^
Cohesiveness	0.3 ± 0.0 ^a^	0.3 ± 0.1 ^a,b^	0.3 ± 0.0 ^b^	0.3 ± 0.0 ^a,b^	0.2 ± 0.0 ^b^	0.3 ± 0.0 ^a,b^
Antioxidant activity DPPH-RSA *	79.4 ± 0.8 ^b^	59.6 ± 1.17 ^c^	87.5 ± 0.9 ^a^	87.9 ± 0.3 ^a^	88.4 ± 0.7 ^a^	88.6 ± 0.7 ^a^

Values are expressed as mean ± SD (*n* = 3). Different lowercase superscripts in the same row indicate significant differences (*p* < 0.05). RSA—radical scavenging activity; DPPH—1,1-diphenyl-2-picrylhydrazine; * μmol TE g^−1^.

**Table 3:** Color attributes of plant-based patties formulated with different concentrations of chia seed powder.

	L*	a*	b*
F1	50.3 ± 0.2 ^a^	11.6 ± 0.5 ^a^	14.7 ± 0.9 ^a^
F2	45.1 ± 0.5 ^c^	11.9 ± 0.1 ^a^	12.6 ± 0.2 ^b^
F3	44.9 ± 0.0 ^c^	5.8 ± 1.2 ^b^	9.5 ± 0.3 ^c^
F4	46.9 ± 0.6 ^b^	5.7 ± 0.2 ^b^	7.5 ± 0.2 ^d^
F5	46.2 ± 0.0 ^b,c^	4.6 ± 0.3 ^c^	7.3 ± 0.1 ^d^
F6	47.0 ± 1.8 ^b^	5.8 ± 0.1 ^b^	9.2 ± 0.3 ^c^

Values are expressed as mean ± SD (*n* = 3). Different lowercase superscripts in the same column indicate significant differences (*p* < 0.05).

**Table 4:** Amino acid composition of the positive control (F2) and selected chia seed powder–fortified. plant-based patty (F5).

Amino Acid (mg/100g)	Positive Control	Best Formulation (F5)
Essential amino acids (EAA)		
Histidine	376.7	404.6
Isoleucine	682.9	794.7
Leucine	1273.6	1481.4
Lysine	977.6	1196.6
Methionine	<200	Nd
Phenylalanine	860.1	964.8
Threonine	591.4	669.2
Valine	836.4	926.3
Tryptophan	167.3	158.2
Non-essential amino acids (NEAAs)		
Alanine	757.0	818.4
Arginine	1295.6	1384.5
Aspartic acid	1959.0	2313.5
Cystine	<200	<200
Glutamic acid	2999	3225
Glycine	687.4	729.1
Proline	572.9	661.3
Serine	894.9	988.8
Tyrosine	428.9	558.1

**Table 5:** Fatty acid composition of the positive control (F2) and selected chia seed powder-fortified plant-based patty (F5).

Fatty Acid (g/100g)	Positive Control	Best Formulation (F5)
Palmitic acid (C16:0)	0.34	0.09
Stearic acid (C18:0)	0.16	0.03
Arachidic acid (C20:0)	0.02	Nd
Behenic acid (C22:0)	0.01	Nd
Saturated fat	0.57	0.14
Cis-9-oleic acid (C18:1n9c)	0.20	0.15
Monounsaturated fatty acid	0.22	0.16
Cis-9,12-Linoleic acid (C18:2n6)	0.02	0.22
Cis-5,8,11,14,17-Eicosapentaenoic acid (C20:5n3)	0.01	Nd
Alpha-Linolenic acid (C18:3n3)	Nd	0.03
Polyunsaturated fatty acid	0.03	0.26
Unsaturated fat	0.25	0.42
Omega 3 (mg/100 g)	14.06	36.88
Omega 6 (mg/100 g)	18.38	217.92
Omega 9 (mg/100 g)	205.97	155.68

## Data Availability

The data presented in this study are available on request from the corresponding author. The data are not publicly available due to privacy restrictions.

## Abbreviations

The following abbreviations are used in this manuscript:

PEM	Protein energy malnutrition
TPP	Textured pea protein
WHC	Water holding capacity
CSP	Chia seed powder
NaCl	Sodium chloride
BP	Black pepper
OP	Onion powder
GP	Garlic powder
BRP	Beetroot powder
PPI	Pea protein isolate
MC	Methyl cellulose
PE	Polyethylene
a_w_	Water activity
TBARS	2-Thiobarbituric acid reactive substances
PCA	Plate count agar
PBC	Psychrophilic bacteria count
TVC	Total viable count
CFU	Colony-forming unit
